# Motion preservation for hyperextension injuries of the cervical spine—an alternative to spondylodesis? A biomechanical cadaver study

**DOI:** 10.1007/s00402-025-05812-0

**Published:** 2025-03-28

**Authors:** Titus Kühlein, Adrian Cavalcanti Kußmaul, Daniela Warnecke, Manuel Kistler, Leandra Bauer, Christopher A. Becker, Wolfgang Böcker, Axel Greiner

**Affiliations:** 1 Department of Orthopaedics and Trauma Surgery, Musculoskeletal University Center Munich (MUM), LMU University Hospital, LMU Munich, Marchioninistr. 15, 81377 Munich, Germany; 2 Orthopedic Research Department, Arthrex GmbH, Munich, Germany; 3 Experimental Orthopaedics, University Hospital Jena, Campus Eisenberg, Waldkliniken Eisenberg, Jena, Germany

**Keywords:** Cervical spine trauma, Biomechanics, Disco-ligamental injury, Minimally invasive, CTDR, ACDF

## Abstract

**Introduction:**

Currently, the gold standard for the treatment of AO type B3 cervical spine injuries is anterior cervical discectomy and fusion (ACDF), leading to an iatrogenic spondylodesis of the affected segment and ultimately bearing the risk of long-term morbidity. This study evaluates the biomechanical properties of a combination of a cervical total disc replacement (CTDR) with anterior fiber tape augmentation for the treatment of AO type B3 injuries in comparison to ACDF.

**Methods:**

14 human cadaveric cervical spine specimens (C5/6) were biomechanically tested under four different conditions: native, after simulation of an AO type B3 injury, after ACDF and CTDR + FiberTape^®^. All conditions were tested in the sagittal, frontal, and transversal plane with a load of 2.25Nm and the dislocation recorded. The mean value of range of motion (ROM) was calculated and analysed to identify differences in ROM and the neutral zone.

**Results:**

In flexion/extension, native testing showed a mean deflection of 11.2° ± 3.3°, the AO type B3 injury of 13.7° ± 2.9°, the ACDF of 6.7° ± 3.8° and the CTDR + tape of 9.3° ± 2.9°. Comparing both the injured specimens to the ACDF group (p < 0.001) and the injured to the tape group (p = 0.005) as well as the native to the ACDF group (p = 0.004), the mean values revealed to be significant. Lateral bending revealed a ROM of 6.8° ± 2.7° in the native, 7.7° ± 2.4° in the injured group, 4.7° ± 2.8° after ACDF, and 5.6° ± 2.4° after CTDR + tape, whereby the injured group values were significantly higher than those after ACDF (p = 0.018). The rotation showed a mean ROM of 5.6° ± 2.8° in the native and 5.8° ± 2.6° in the injured group, 4.0° ± 2.1° after ACDF and 6.3° ± 2.8° after CTDR + tape, without significant differences.

**Conclusion:**

The combination of a CTDR + FiberTape proved to stabilize AO type B3 cervical spine injury adequately in the most compromised sagittal plane while maintaining micro-mobility and approaching physiological segment mobility.

## Introduction

Hyperextension injuries belong to the most frequent injuries of the cervical spine and are usually caused by high-impact trauma, such as traffic accidents or frontal collisions. The concomitant rise in the elderly society, has led to an increase in hospitalization rate of patients with neck injuries, such as hyperextension injuries, here however primarily based on low-impact trauma [1, 2].

According to the Association of Osteosynthesis (AO) classification, hyperextension injuries can be classified as type B3 injuries, including the rupture of the anterior longitudinal ligament (ALL) and commonly compromising the intervertebral disc [3, 4].

Even though relevant cervical separation is prevented, given the integrity of the posterior hinge [3, 4], these injuries bear the risk of cervical destabilization and neurological damage or disc extrusion [5], ultimately demanding surgical stabilization [6].

Currently, surgical treatment of anterior tension band injuries consists of anterior cervical discectomy and fusion (ACDF) [7].

If one considers the pathophysiology of anterior tension band injuries, instabilities mainly occur in the sagittal plane: As demonstrated by Stein et. al, a hyperextension injury increases the mobility in the sagittal plane by 90%, whereas in lateral bending and axial rotation only by 27% and 21%, respectively [5].

ACDF significantly nullifies the mobility of the cervical spine in all motion planes, impeding the physiological mobility of the cervical spine [7].

Alternatively to this established rigid osteosynthesis method, minimally invasive tape suture constructs have been successfully implemented in knee, ankle, pelvic and shoulder surgery striving for motion preservation in traumatically affected motion segments [8–14]. These constructions aim to imitate the original function of the damaged ligament and thus restrict the movement of a joint mainly in one plane when it is subjected to tension and consequently reducing the impact on the joint to a minimum.

Regarding an impaired cervical disc, the idea of motion preserving disc replacement is already established in form of cervical total disc replacement (CTDR) [15, 16]. Indications for this procedure in the cervical spine include radiculopathy or myelopathy caused by either one or two levels of anterior cervical compression and degenerative disc disease (DDD), however it is currently not authorized for treatment in cases of traumatic instability [17, 18].

A prior study on synthetic cervical spine models first investigated the idea of motion preservation with a tape suture augmentation as a treatment for traumatic ALL injuries, like AO type B3 injuries, with promising biomechanical results [4]. In this study, the tape construction was not combined with a prothesis such as in CTDR, but with the same cage that was used for the comparative procedure of ACDF. The results revealed that ACDF and Cage + Tape showed no significant difference in flexion/extension (p = 0.146), whereas there was significantly more movement of the tape construction in lateral bending (p = 0.013) and rotation (p < 0.01). [4].

Therefore, this study aims to combine tape suture constructs (tape) with CTDR to add up their advantages in motion preservation whilst ensuring the treatment of the underlying instability. Their biomechanical properties are evaluated in cadaver specimens and compared to physiological conditions, AO type B3 injury status and to ACDF.

## Materials and methods

16 human cadaveric cervical spine specimens were used in this biomechanical in vitro study. Their use in this study was approved in advance by the ethical committee of the LMU Munich (EA 20–1128). The specimens were stored frozen at < − 20 °C until being thawed at room temperature one day prior to their experiments.

The handling and experimental testing of the specimens was performed in accordance with the established testing criteria for spinal implants published by Wilke et al. [19]. Exclusion criteria contained: donors age over 80 years, cancer with known bone infiltration, leukemia, tuberculosis, specimens with any injury to the cervical spine [19]. Furthermore specimens were analyzed according to radiological criteria.

Therefore, Computed tomography (CT) scans of every specimen were performed prior to their dissection to identify medical preconditions concerning the intervertebral joints. The exclusion criteria and concomitant suitability for the study was evaluated in collaboration with a radiology specialist, leading to the exclusion of specimen no. 1 and 6 due to advanced degeneration and local ossification in the C5/6 region. Consequently, leaving 14 specimens to undergo testing.

After thawing, the C5/6 segment of each specimen was isolated. All surrounding soft tissue was dissected while keeping the ligaments, cervical discs and vertebrae intact for experimental testing.

**Fig. 1:**
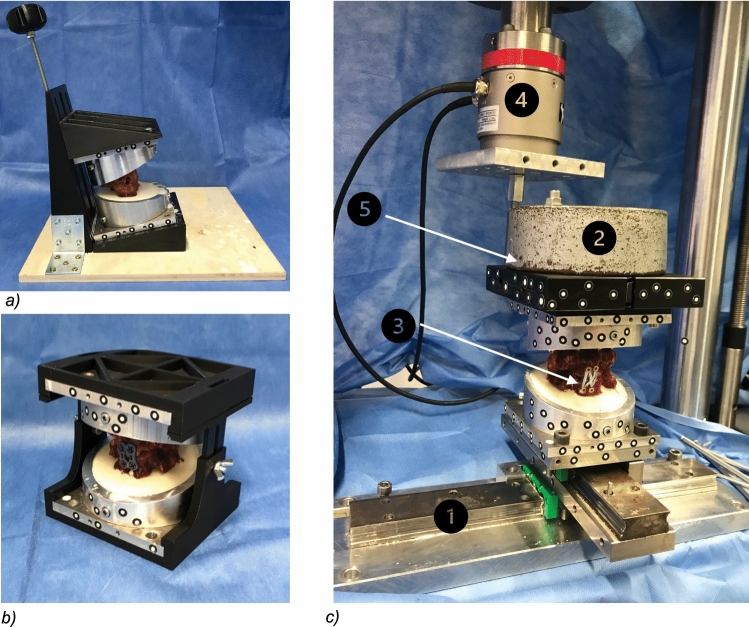
**a** specimen embedding using a special designed casting guide, **b** implementation of ACDF using a fixation tool to maintain physiological alignment, **c** apparatus for testing with 1: x/y table, 2: preload 50 N, 3: specimen, 4: testing machine, 5: lever arm 15 cm

The specimens were then casted into specially designed pots and embedded in resin (RenCast^®^ FC 52/53 Isocyanate/FC 53 Polyol, Huntsman Corporation^®^, Salt Lake City, UT, USA), maintaining their physiological alignment (Fig. 1a).

**Fig. 2:**
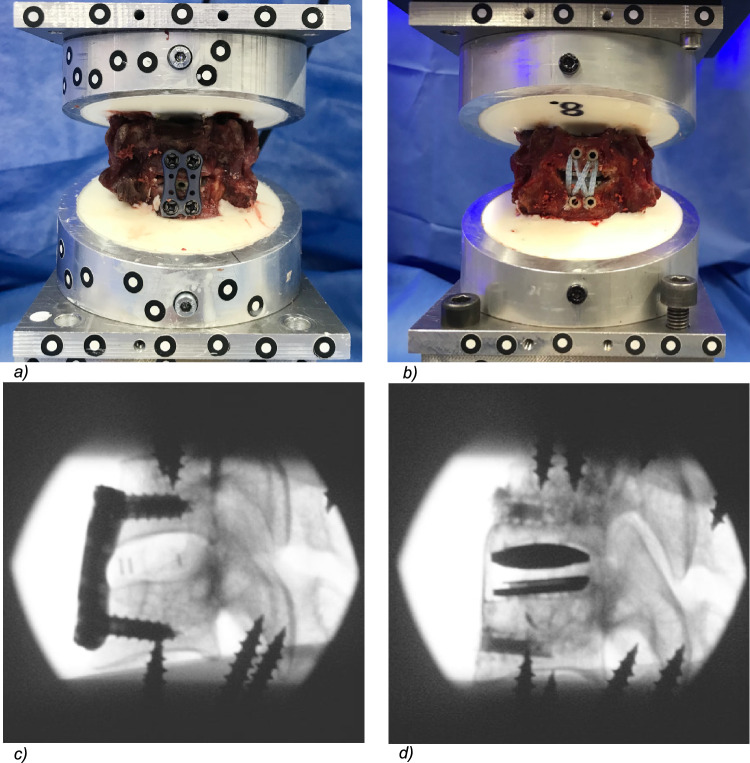
Specimens provided with ACDF (**a**) and CTDR + FiberTape^®^ (Arthrex, Naples, FL, USA) (**b**) as well as x-rays of specimen provided with ACDF (**c**) and CTDR + FiberTape^®^ (**d**); screws were inserted into the upper and lower edge of the specimen to increase the stability of the embedding

In accordance with the method used by Röhl et al., screws were placed onto the upper and lower surfaces of the specimens before embedding to ensure optimal positioning and to provide sufficient stability and force transmission (Fig. 2) [20].

Additionally, the pots were previously beveled by 13° to receive sufficient space for internal fixation and to add stability by casting the processus spinosus. The casting guide was designed using CAD-software (program: Fusion 360^®^ Autodesk, San Rafael, CA, USA) and 3D printed (3D-printer: Original Prusa i3MK3S + ^®^, Prusa Research, Prague, Czech Republique) to obtain an exact 13° casting line (Fig. 1a).

The applied biomechanical loading method was adapted from a protocol of Röhl et al., which overall comprises a sequential testing, imitating isolated head movement. It consists of flexion–extension, lateral bending to the right and left side and axial rotation to the right and left, respectively [20].

In this study, pure moments for flexion–extension and lateral bending were applied by the all-electric testing machine (Instron e10000, Norwood, MA, USA) via a 15 cm lever arm fixed to the specimen holder, consisting of a telescopic rail with ball bearings to ensure pure bending moment during motion (Fig. 1c) [21].

To avoid unphysiological tension during the mounting process, the base was set on a x/y table. To additionally achieve physiological conditions in terms of preloading and to simulate the head weight of approximately 5 kg, an initial weight of 50 N was applied on the upper pot [22]. During rotational testing, the testing machine directly applied axial rotation momentum as well as the corresponding preload[20].

Fifteen cycles were performed for each of the three loading scenarios (flexion/extension, lateral bending and axial rotation) to ensure final implant adaption and to achieve reproducible results. The loading oscillated around neutral position to simulate complete head movements [19]. The maximum speed was set to 5°/s [19] and the momentum was set to 2.25 Nm, which was provided via the lever arm with a force of 15 N.

An optical measurement system (GOM Aramis 3D Camera 12 M, GOM GmbH, Braunschweig, Germany) allowed the exact measurement of the bending angles (°) of the specimens with a frequency of 3–5 Hz. Data of the optical measurement system was used to calculate the range of motion (ROM) in the sagittal and frontal plane, whereas data from the testing machine provided the ROM for the rotation.

Furthermore, the testing machine recorded changes in force and displacement, allowing detailed analysis of the motion, such as the neutral zone.

The neutral zone, described by Panjabi et al. as the portion of the spinal motion load–deflection curve where motion is produced with a minimal resistance, was calculated from the force–displacement-diagram provided by the testing machine [23]. A specially programmed code in MATLAB (Version R2022a, MathWorks Inc., Natick, MA, USA) was used to determine stiffness thresholds and calculate the neutral zone [24].

Overall, every specimen underwent 4 Testing cycles: First, initial testing in native condition was performed. After an AO type B3 injury was created by the entire transection of the ALL and intervertebral disc [3], the specimens underwent the testing protocol again. Lastly, an anterior discectomy was performed, and the specimens were treated with first ACDF and then CTDR + Tape.

In case of ACDF, a disc replacement (DePuy Synthes Cervios Cage curved, size 5 mm, West Chester, PA, USA) was implanted with a titanium anterior cervical plate (DePuy Synthes^®^ 14 mm) fixed with 4 screws (DePuy Synthes^®^ ø 4.0 mm, L 16 mm) [25] (Fig. 2a, c).

After testing the ACDF, the disc cage was replaced by a disc prothesis (ProDisc C Vivo^®^, Centinel Spine, Inc., West Chester, Pennsylvania). Implants of size 5 and 6 were used after determining the intervertebral disc space radiographically. To implement the tape fixation, the preexisting screw holes on the ventral side of the vertebral bodies were used and 4 PEEK SwiveLock^®^ ø 4.75 mm anchors equipped with two tapes (FiberTape^®^ or TigerTape^®^, both Arthrex, Naples, FL, USA) were inserted using the double row technique (Fig. 2b, d). During implant placement, both pots were again fixed in the custom-made 3D printed guide to ensure a physiological alignment (Fig. 1b).

Statistical analysis was performed with IBM SPSS Statistics^®^ version 29 (Armonk, NY, USA). Firstly, normal distribution was asserted using the Kolmogorov–Smirnov test (p > 0.05), as well as the homogeneity of variances using Levene’s test which showed that equal variances could be assumed in all tested planes (p > 0.05),. The ROM of the remaining specimens was evaluated by means of a single-factor analysis of variance in which the mean values of the individual fittings of a specimen were compared with each other. The analysis of variance (ANOVA) was performed using the post-hoc test (Bonferroni) to identify differences in ROM and the neutral zone. The level of significance was set at p ≤ 0.05.

## Results

**Fig. 3:**
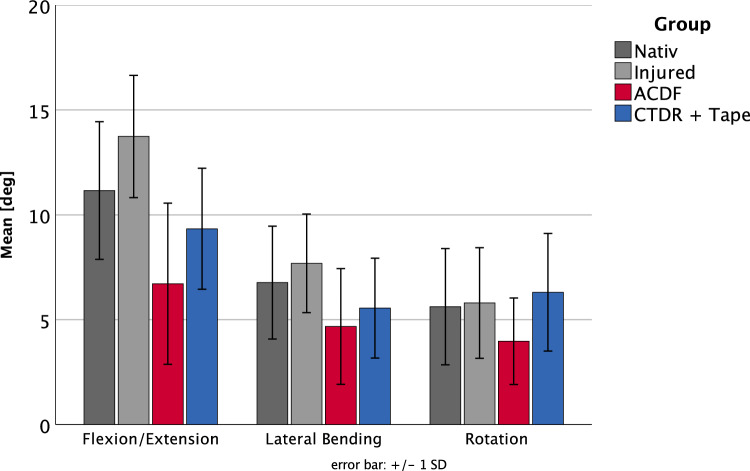
Mean ROM values of the four tested conditions: native (dark grey), injured (light grey), ACDF (red), and FiberTape^®^ + CTDR (blue) obtained for flexion/extension, lateral bending, and axial rotation

The average age of the donors was 58.5 years with a maximum age of 76 years and a minimum age of 26 years. The sex distribution showed 11 males and 5 females (Table 2). Regarding flexion/extension, native tests showed a mean deflection of 11.2° ± 3.3°, the AO type B3 injury of 13.7° ± 2.9°, the ACDF of 6.7° ± 3.8° and the CTDR + Tape of 9.3° ± 2.9° (Fig. 3, Table 1, Table 2). The mean values revealed to be significant when comparing the injured specimens to the ACDF group (p < 0.001) and the injured to the tape group (p = 0.005) as well as the native to the ACDF group (p = 0.004) (Table 1, Fig. 3, Fig. 5) in the sagittal plane.

**Table 1:** ANOVA Results of the statistical analysis (ANOVA) of ROM and NZ both in (°) and standard Deviation (SD), **bold** highlights significant findings (p < 0.05)

	ROM [°] mean ± SD	p < 0.05					p < 0.05			
		Native	Injured	ACDF	CTDR + tape	NZ [°] mean ± SD	Native	Injured	ACDF	CTDR + tape
Flexion/extension										
Native	11.2 ± 3.3		0.172	**0.002**	0.853	0.046 ± 0.055		0.543	**0.004**	1.0
Injured	13.7 ± 2.9	0.172		** < 0.001**	**0.003**	0.016 ± 0.000	0.543		** > ** **0.001**	0.289
ACDF	6.7 ± 3.8	**0.002**	** > ** **0.001**		0.135	0.109 ± 0.060	**0.004**	** > ** **0.001**		**0.009**
CTDR + tape	9.3 ± 2.9	0.853	**0.003**	0.135		0.051 ± 0.047	1.0	0.289	**0.009**	
Lateral bending										
Native	6.8 ± 2.7		1.0	0.286	1.0					
Injured	7.7 ± 2.4	1.0		**0.028**	0.277					
ACDF	4.7 ± 2.8	0.286	**0.028**		1.0					
CTDR + tape	5.6 ± 2.4	1.0	0.277	1.0						
Rotation										
Native	5.6 ± 2.8		1.0	0.526	1.0					
Injured	5.8 ± 2.6	1.0		0.372	1.0					
ACDF	4.0 ± 2.1	0.562	0.372		0.096					
CTDR + tape	6.3 ± 2.8	1.0	1.0	0.096						

Considering lateral bending, the mean ROM was 6.8° ± 2.7° in the native group, 7.7° ± 2.4° in the injured group, 4.7° ± 2.8° after ACDF and 5.6° ± 2.4° after CTDR + Tape (Fig. 3). The statistical analysis showed that the mean values of the injured group were significantly higher (p = 0.018) than those after ACDF (Table 1, Fig. 3).

The rotation showed a mean ROM of 5.6° ± 2.8° in the native group, 5.8° ± 2.6° in the injured group, 4.0° ± 2.1° after ACDF and 6.3° ± 2.8° after CTDR with tape (Fig. 3). Here, no significant differences were found in the single factor analysis (Table 1, Fig. 3).

**Fig. 4:**
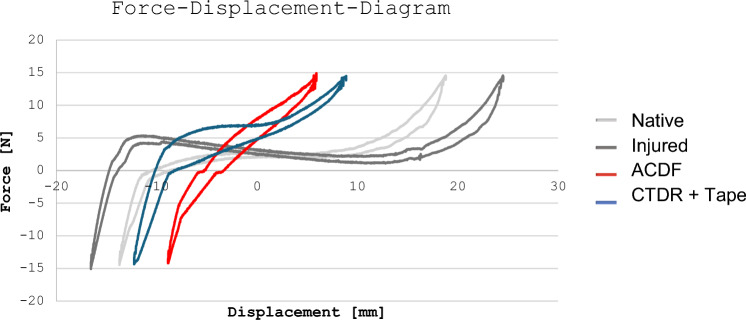
Exemplary display of one tested cycle under all testing conditions of specimen nr.5. This diagram shows the movement of the actuator of the testing machine when testing flexion/extension and thereby moving up and down. Positive force values refer to a tractive power on the actuator, indicating a flexion movement of the specimen. Negative force values refer to compressive power on the actuator indicating an extension movement of the specimen. Values of the displacement should be considered as relative instead of absolute as the neutral position varies between the different testing conditions

**Fig. 5:**
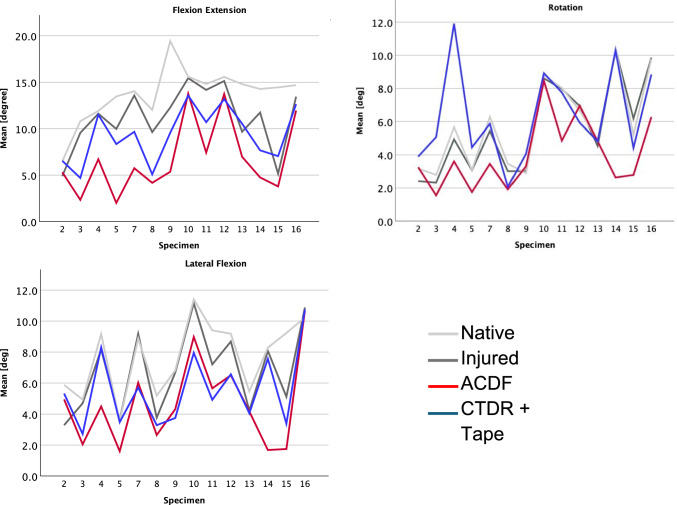
Mean ROM in the three tested planes with the four tested conditions: native (dark grey), injured (light grey), ACDF (red), and FiberTape^®^ + CTDR (blue) obtained for flexion/extension, lateral bending, and axial rotation

To gain a deeper insight into the dynamics of the systems, the neutral zone as stiffness parameter was calculated from the force–displacement diagram of the sagittal plane: The native condition testing revealed a neutral zone of 0.046° ± 0.055°, the injured of 0.016° ± 0.0°, the ACDF of 0.109° ± 0.060° and the tape of 0.051° ± 0.047 (Table 1). Regarding the NZ, a positive correlation to the stiffness of the construct could be observed. Here, the ACDF group showed a significantly higher stiffness with a NZ of 0.11° ± 0.06°, approximately twice as high as the native (p = 0.004) or tape group (p = 0.009) and nearly 7 times higher than the injured group (p < 0.001) (Table 1) (Fig. 4). Around the neutral position, slight movement is possible without interference of the implant as pictured in Fig. 4. The moment the implant is loaded with tension, the resistance rises quickly. In contrast, the FiberTape^®^ construct accomplished a constant but slower rise in resistance and eventually reached the stiffness threshold quicker than the ACDF (Fig. 4).

## Discussion

The treatment of traumatic hyperextension injuries of the lower cervical spine, mainly consisting of AO type B3 injuries, has been standardized for many years using ACDF [26, 27]. In this study, a novel combination of CTDR and FiberTape^®^ is being tested and compared to ACDF.

Both techniques, the ACDF (p < 0.001) and the FiberTape^®^ augmented CTDR (p = 0.005) significantly reduced the extension of the injured cervical segment. However, only the FiberTape^®^ construct did not differ significantly from the native condition regarding the maximum dislocation (p = 0.853). This illustrates the biomechanical stability of the FiberTape^®^ while preventing an iatrogenic arthrodesis and approaching physiological motion limits (Table 1).

As expected, no significant difference was found between FiberTape^®^ construct, ACDF and native condition for lateral bending and rotation as these planes are less affected by a hyperextension injury (p > 0.05) [5]. However, a tendency towards the lowest ROM with ACDF could be observed. In contrast to the plate ostheosynthesis that restricts movement both in tension and in compression, the tape relaxes when it is not under tension. Due to the symmetrical paracentral course of the tapes in the double-row method, only one part of the tape construct is tensed during both lateral flexion and rotation, while the other part relaxes. As the tape fixation by means of anchors allows a certain amount of leeway for changes in direction, the tape under tension permits more movement when tilting or rotating to the right and left only by changing its angle, thus reducing the stiffening and creating a dynamic and force depended joint limitation.

Contrary to the expectations, there is still a lot of motion in the sagittal plane of the effected segment after ACDF (6.7° ± 3.8°) even though significantly lower than the native (p = 0.004) and injured (p < 0.001) condition. These values appear relatively high for an osteosynthesis that is considered to be rigid. Stein et al. compared ACDF with an integrated interbody device in cadaveric cervical spine specimens. Flexion + extension of the C5/6 segment supplied with ACDF showed an average ROM of 5.0° in the sagittal plane. Given that a moment of 1.5Nm was used in their study, compared to 2.25Nm in this one, the values seem comparable [28].

Yet, since the combination with a cervical cage is not suitable for motion, this may explain the high rate of pseudarthrosis after ACDF [29]. According to a study by Liu et al., the pseudarthrosis rate lies between 5 and 10% for single level anterior cervical discectomy and fusion procedures [30]. As the movement after CTDR lies in the intended joint of the prosthesis, the pseudarthrosis rate is lower than after ACDF [31].

The here performed AO type B3 fracture displayed an increase in the ROM in all planes when compared to the native group, even though not significantly (p > 0.05). Yet, as the test apparatus reached its extension limits at around 15° and torques of 2 Nm could not be achieved in all specimens due to massive instability, the values of the injured group in the sagittal plane are presumably higher than measured in this experimental set-up (13.7° ± 2.9°), underlining the expectations of an instable sagittal plane after a hyperextension trauma (Table 1). Therefore, the present insignificance regarding hyperextension is based on mechanical reasons. From a clinical point of view, a highly relevant instability and concomitant significant difference can be assumed. Also, these results confirm the lateral bending and axial rotation to be less compromised in an AO type B3 injury since the posterior longitudinal ligament and facet capsules are mostly intact, providing resistance to disruption and stabilization of the frontal and transversal plane [32].

To obtain detailed information about the movement and rigidity of the intervertebral joint, the neutral zone (NZ) was calculated for all specimens in the sagittal plane. As the sagittal plane is the most affected plane in an AO type B3 injury, the focus was set only on this plane. In this study, the stiffness threshold technique was implemented [24]. So far no recommendation exists on how to exactly calculate the NZ and the use of several techniques may lead to different results [24]. The results of the NZ and the fact that the plate provided specimen shows a less smooth bending curve reflect the rigidity of the plate related ACDF construction (Table 1) (Fig. 4). Furthermore, the approximation of the NZ from the tape to the native group can be interpreted as an approximation to physiological motion in the joint. This is also illustrated by the bending curves in Fig. 4.

It needs to be noted, that the movement of the specimens during loading (exemplary shown in Fig. 4), did not always originate from the same starting point (neutral position). This is based on both the architecture of the setup and the alterations of the neutral position after inserting the implants. Consequently, when interpreting Fig. 4, the relevance lays in the form of the graphs, not in their absolute numbers: While the injured condition displays a longer horizontal and even fluctuating course, reflecting instability, the CTDR + FiberTape^®^ approximates physiological conditions, yet providing more stability (shorter horizontal course) compared to the native state. On contrary, the ACDF displays an almost linear force–displacement course, representing an almost direct force transmission on the implant, ultimately underlining its stiffness.

The rigidity of the system may not be relevant for the explanation of short-term complications of ACDF, the most frequent including mal-positioning with nerve root irritation, implant loosening, plate and screw breakage, screw-induced triangular fractures, and soft tissue irritation, especially of the esophagus [25, 29]. In a study of Ning et al., the total complication rate in 2233 examined cases after ACDF was 10.7% including revision surgery [29].

Considering the long-term consequences of ACDF on the other hand, the rigid fusion of one or more vertebral segments often leads to accelerated degeneration of the adjacent segment, which is widely known as adjacent segment disease (ASD) [33]. Common conditions are disc degeneration, disc herniation, instability, spinal stenosis, spondylosis, and facet joint arthritis [34]. These ultimately may lead to radiculopathy. According to a study by Hilibrand et al., the probability of developing a nerve root irritation lies within the range of 2–3% per year [35]. Although the underlying pathophysiology has not yet been finally understood, there is good evidence suggesting that the cause is suspected to be due to an increase in motion and pressure at the adjacent segments [36]. Eck et al. demonstrated a significant pressure enhancement in segments adjacent to with ACDF treated segments [36]. This was found to be 73.2% in the C4/5 segment and 45.3% in the C6/7 segment after C5/6 fusion [36]. It is postulated that the increase in pressure leads to an acceleration of the normal aging process of discs, due to changes in the metabolism. [36].

The need for an alternative treatment to ACDF with the aim to maintain the function of the cervical spine as the most mobile part of the human spine [37], has become more evident.

Recently, Dufour et al. demonstrated that motion preserving using CTDR is feasible in the cervical spine and found significant evidence that it can reduce ASD[31]. A total of 384 patients underwent 535 CTDR procedures and 72.1% of the implants kept their mobility at the 2-year follow-up. Segmental motion was increased to an average of 8° from 6° preoperatively and ASD occurred in only 2.9% of patients at 5 years [31]. Furthermore, studies found that the likelihood of secondary surgery was 50% lower in the long-term follow-up with CTDR compared to ACDF [38].

Nevertheless, complications are also known using CTDR: As with all anterior approaches to the cervical spine, there is a risk of postoperative dysphagia and vertebral artery injuries [38]. However, according to a metanalysis of 3223 patients, both were observed significantly less frequent than with ACDF [38]. Furthermore, there is a risk of heterotopic ossification (HO): In this case, uncontrolled bone growth occurs, which in advanced stages can lead to arthrodesis of the joint [15, 16, 38]. Research is still ongoing determining the cause, but changes in the biomechanics of the cervical spine due to the implant are discussed as potential causes [15]. With increasing limitation of motion, CTDR approaches the biomechanical characteristics of ACDF and thus also carries the risk of ASD. However, the risk is significantly lower than after ACDF and lies at 7.3% at midterm follow-up to 5 years in a metanalysis [38].

Regarding the limitations, this study found that the natively measured ROM was significantly lower than the one of a reference study of Anderst et al.: Here, the authors analized three-dimensional intervertebral kinematics in healthy young adult cervical spines during dynamic functional loading using biplane radiographs [39]. When comparing their results to the native testing results of this study, the flexion/extension in this study showed to be 11.2° ± 3.3° (reference 19.7°), the lateral bending 6.8° ± 2.7° (reference 12.3°) and the rotation 5.6° ± 2.8° (reference 9.3°) (Table 1) [39]. These differences can mostly be explained by a higher average donor age of 58.5 years in this study compared to an average age of 27.4 years in the reference study, as the pre-traumatic physiological ROM is considered to be dependent on the age of the patient (Table 2) [39]. Yet, considering the increasing incidence of the injury in the elderly population, this study may approach the current clinical appearance more adequately.

Another limitation regarding the average donor age is that according to Dufour et al. the ideal age for successful CTDR is in the 4th decade [31]. The average age of the specimens in this study was 58.5 years. It must be added, however, that the suitability refers not only to the postoperative segmental ROM but also in particular to the long-term consequences. These do not affect an ex vivo study but must be considered for a later clinical application.

Furthermore, freezing, storing and thawing of the specimens is known to induce alterations in ligamentous tissue and consequently limits the comparison to in vivo measurements [40]. Hence, the transmission of implant provided test results into clinical reality is limited. It should also be acknowledged that the uncertainty of the measurement method has not been assessed. Nevertheless, the relation of the single tests to each other have the same underlying confounder, such as preload, time in the freezer, loading etc., and might approach in vivo relations.

Furthermore, all tests are performed at the point zero, meaning directly after the intervention. An ACDF aims to fuse two segments without any remaining motion, which is initiated through the plate osteosynthesis but brought to a complete fusion through scarring as shown in the in vivo study on goats by Peterson et al. [41]. Naturally this is not the case when using cadaver specimen and might be an explanation why the ROM of the ACDF group revealed to be so high. Adding the natural healing process may therefore increase the difference in ROM between the two treatment options after some time with a non-moving ACDF and a free movable CTDR + Tape fusion.

Finally, only 14 specimens were tested in this study, yet compared to similar studies it lays within the normal range [20]. Still, larger studies are necessary to obtain precise results and a better understanding of the procedure.

## Conclusion

This study was able to prove the feasibility of the concept of motion preservation for the treatment of AO type B3 injuries with a FiberTape^®^ construct in combination with a CTDR. It could be shown that this new/alternative treatment strategy allows sufficient biomechanical stabilization of the compromised sagittal plane while maintaining micro-mobility and approaching physiological segment mobility.

## Appendix

See Fig. 5 and Table 2

**Table 2:** Demographics of the specimens

#	Age	Height [cm]	Weight [kg]	BMI	Sex	Primary cause of death	Secondary cause of death	Bone/joint
1	61	177,8	106,12	33,57	Male	Hypertensive and atherosclerotic cardiovascular Disease	–	Arthritis
2	70	175,26	82,99	27,02	Male	Acute cardiopulmonary arrest alcoholism	Failure to thrive, orthostatic hypotension, urinary retention	Osteoarthritis
3	71	175,26	110,66	36,03	Male	Acute hypoxic respiratory failure exacerbation of chronic obstructive pulmonary disease	Idiopathic pulmonary fibrosis	Osteoarthritis
4	76	165,1	93,42	34,28	Female	Acute hypoxic respiratory failure aspiration pneumonia,	Acute cerebrovascular accident, Standford A	–
5	51	182,88	97,05	29,02	Male	Complication of blunt force trauma, probable fall	Chronic alcohol abuse, chronic anticoagulation	–
6	72	177,8	75,74	23,96	Male	Cardiorespiratory arrest alzheimer's disease	–	Deformity in spine
7	29	200,66	79,82	19,83	Male	Acute brain injury, cardiogenic shock pneumonia	Sudden cardiac arrest, schizophrenia, autism	–
8	52	175,26	110,20	35,88	Male	Acute hypoxemic respiratory failure acute upper Gastrointestinal bleed	Acute encephalopathy, alcoh	–
9	74	147,32	69,84	32,18	Female	Hemorrhagic cerebrovascular accident	–	–
10	68	162,56	87,07	32,95	Female	Coronary artery disease, COPD	–	Osteoarthritis
11	68	167,64	58,50	20,82	Female	CAD, HTN, Genetics, Lifestyle	–	–
12	65	180,34	122,90	37,79	Female	Cardiorespiratory arrest	Chronic obstructive pulmonary disease, hypertension	History of falls
13	70	185,42	131,97	38,39	Male	Non-alcoholic steatohepatitis, pleural effusion	Portal hypertension	Arthritis
14	37	193,04	88,89	23,86	Male	Sudden unexpected death in epilesy	–	–
15	46	170,18	122,45	42,28	Male	Hypertensive and atherosclerotic cardiovascular Disease,	Obesity, reactive airway disease	–
16	26	185,42	124,72	36,28	Male	Acute combined methadone and ethanol toxicity	–	–
